# *O*,*O*′-Dimethyl (cyclo­hexyl­amido)­thio­phosphate

**DOI:** 10.1107/S160053681203766X

**Published:** 2012-09-08

**Authors:** Fahimeh Sabbaghi, Mehrdad Pourayoubi, Marek Nečas

**Affiliations:** a Department of Chemistry, Zanjan Branch, Islamic Azad University, Zanjan, Iran; b Department of Chemistry, Ferdowsi University of Mashhad, Mashhad, Iran; c Department of Chemistry, Faculty of Science, Masaryk University, Kotlarska 2, Brno CZ-61137, Czech Republic

## Abstract

The P atom in the title compound, C_8_H_18_NO_2_PS, is bonded in a distorted tetra­hedral PSO_2_N environment with bond angles in the range of 99.23 (5)–115.17 (4)°. The cyclo­hexane ring is disordered over two sets of sites with refined occupancies of 0.528 (5) and 0.472 (5). The ring in both disorder components adopts a chair conformation with the N—H group oriented equatorially. In the crystal, pairs of P=S⋯H—N hydrogen bonds form inversion dimers.

## Related literature

For related structures, see: Chivers *et al.* (2003 ▶); Balazs *et al.* (1999 ▶); García-Hernández *et al.* (2006 ▶). For compounds with a P(S)(N)(O)_2_ skeleton, see: García-Hernández *et al.* (2006 ▶). For the distorted tetra­hedral configuration of phospho­r­amidates and their chalco-derivatives, see: Rudd *et al.* (1996 ▶).
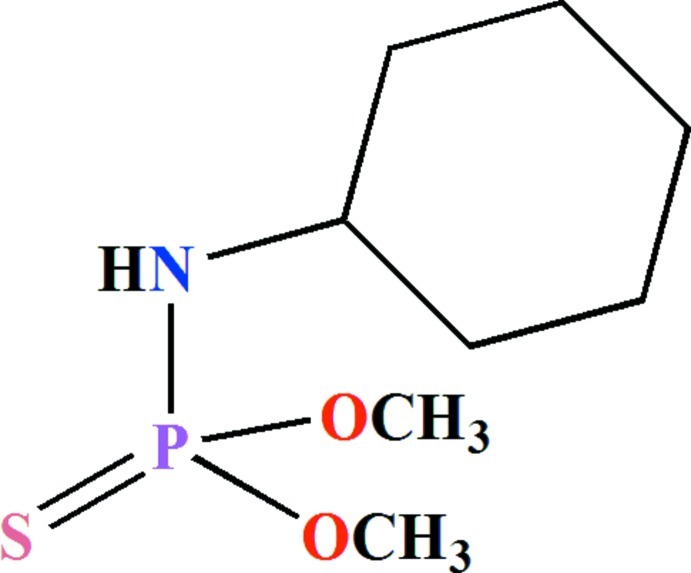


## Experimental

### 

#### Crystal data

C_8_H_18_NO_2_PS
*M* *_r_* = 223.26
Triclinic, 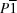

*a* = 6.5214 (4) Å
*b* = 9.2078 (6) Å
*c* = 10.5763 (7) Å
α = 67.447 (6)°
β = 80.212 (5)°
γ = 78.863 (5)°
*V* = 572.24 (6) Å^3^
*Z* = 2
Mo *K*α radiation
μ = 0.40 mm^−1^
*T* = 120 K
0.50 × 0.50 × 0.30 mm

#### Data collection

Oxford Diffraction Xcalibur Sapphire2 diffractometer
Absorption correction: multi-scan (*CrysAlis RED*; Oxford Diffraction, 2009 ▶) *T*_min_ = 0.942, *T*_max_ = 1.000
3413 measured reflections
2017 independent reflections
1731 reflections with *I* > 2σ(*I*)
*R*_int_ = 0.011

#### Refinement

*R*[*F* ^2^ > 2σ(*F* ^2^)] = 0.025
*wR*(*F* ^2^) = 0.069
*S* = 1.07
2017 reflections
170 parameters
163 restraints
H atoms treated by a mixture of independent and constrained refinement
Δρ_max_ = 0.21 e Å^−3^
Δρ_min_ = −0.33 e Å^−3^

### 

Data collection: *CrysAlis CCD* (Oxford Diffraction, 2009 ▶); cell refinement: *CrysAlis CCD*; data reduction: *CrysAlis RED* (Oxford Diffraction, 2009 ▶); program(s) used to solve structure: *SIR92* (Altomare *et al.*, 1993 ▶); program(s) used to refine structure: *SHELXL97* (Sheldrick, 2008 ▶); molecular graphics: *Mercury* (Macrae *et al.*, 2008 ▶); software used to prepare material for publication: *SHELXTL* (Sheldrick, 2008 ▶) and *enCIFer* (Allen *et al.*, 2004) ▶.

## Supplementary Material

Crystal structure: contains datablock(s) I, global. DOI: 10.1107/S160053681203766X/lh5513sup1.cif

Structure factors: contains datablock(s) I. DOI: 10.1107/S160053681203766X/lh5513Isup2.hkl

Additional supplementary materials:  crystallographic information; 3D view; checkCIF report

## Figures and Tables

**Table 1:** Hydrogen-bond geometry (Å, °)

*D*—H⋯*A*	*D*—H	H⋯*A*	*D*⋯*A*	*D*—H⋯*A*
N1—H1*N*⋯S1^i^	0.791 (16)	2.695 (17)	3.4633 (13)	164.3 (14)

Symmetry code: (i) 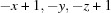.

**Fig. 1.**
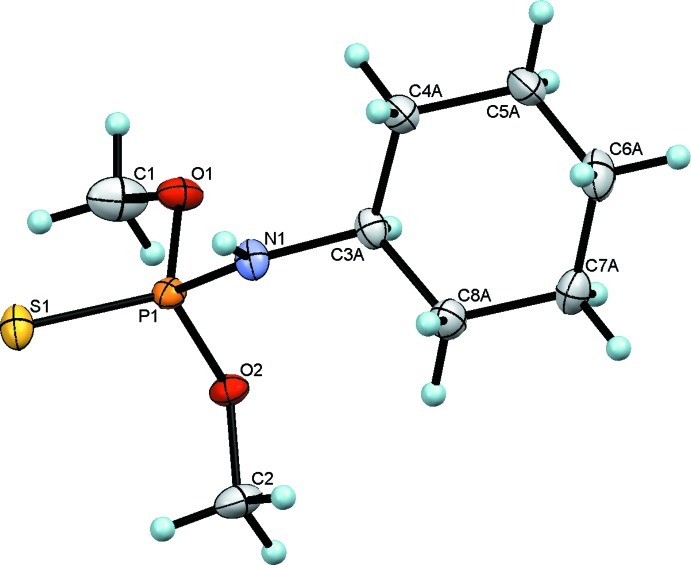
The molecular structure of the title compound with ellipsoids shown at the 50% probability level and H atoms are drawn as small spheres of arbitrary radii. The minor component of disordered part has been omitted for clarity and only one orientation is shown for the disordered cyclohexyl group.

**Fig. 2.**
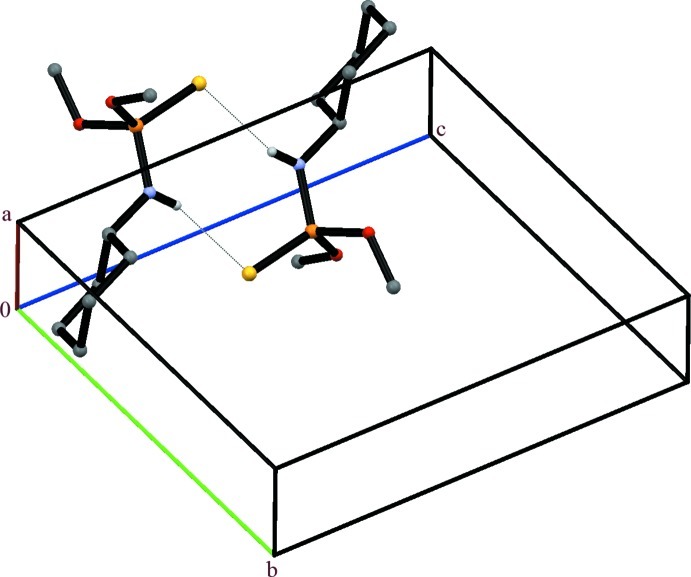
Pair of P═S···H—N hydrogen bonds (shown as dotted lines) in the hydrogen-bonded dimer. The H atoms not involved in hydrogen bonding have been omitted for clarity.

## supplementary crystallographic information

### Comment

The structure determination of the title compound (Fig. 1) was performed as a part of a project on the synthesis of a new phosphorus(V)-nitrogen compound belonging to the family of phosphoramidothioate with potential applications as S-donor ligands, similar to those observed for reported compounds with a P═S bond (Chivers *et al.*, 2003; Balazs *et al.*, 1999; García-Hernández *et al.*, 2006).

The P═S (1.9351 (5) Å), P—O (1.5823 (10) and 1.5853 (9) Å) and P—N (1.6153 (12) Å) bond lengths are within the expected values for compounds with a P(S)(N)(O)_2_ skeleton (García-Hernández *et al.*, 2006).

The P atom has a distorted tetrahedral configuration (Fig. 1) as it has been noted for phosphoramidates and their chalco-derivatives (Rudd *et al.*, 1996). The bond angles at the P atom vary in the range 99.23 (5) [O1—P1—O2] to 115.17 (4)° [O1—P1—S1]. The C—O—P bond angles are 120.01 (9) [C1—O1—P1] and 119.12 (9)° [C2—O2—P1]. In the crystal, inversion dimers are formed by pairs of P═S···H—N hydrogen bonds, Table 1 and Fig. 2.

### Experimental

To a solution of [CH_3_O]_2_P(S)Cl (1.7 mmol) in dry CH_3_CN (30 ml), a solution of cyclohexylamine (3.4 mmol) in the same solvent (5 ml) was added at ice bath temperature. After 4 h stirring, the solvent was removed and the product was washed with distilled water and recrystallized from methanol at room temperature. The single crystals, suitable for X-ray analysis were obtained from this solution after a few days at room temperature.

### Refinement

All carbon bound H atoms were placed at calculated positions and were refined as riding with their *U*_iso_ set to either 1.2*U*_eq_ or 1.5*U*_eq_ (methyl) of the respective carrier atoms; in addition, the methyl H atoms were allowed to rotate about the C—C bond. Nitrogen bound H atom was located in a difference Fourier map and refined isotropically. The disordered cyclohexyl group was modeled over two sites using similarity restraints on anisotropic displacement parameters. To maintain a correct hydrogen geometry, a dummy atom with zero occupancy was created and constrained to share the same site (EXYZ) and anisotropic displacement parameters (EADP) with a fully occupied carbon atom bound to N1.

### Crystal data

**Table:**

C_8_H_18_NO_2_PS	*Z* = 2
*M**_r_* = 223.26	*F*(000) = 240
Triclinic, *P*1	*D*_x_ = 1.296 Mg m^−^^3^
Hall symbol: -P 1	Mo *K*α radiation, λ = 0.71073 Å
*a* = 6.5214 (4) Å	Cell parameters from 2495 reflections
*b* = 9.2078 (6) Å	θ = 3.2–27.7°
*c* = 10.5763 (7) Å	µ = 0.40 mm^−^^1^
α = 67.447 (6)°	*T* = 120 K
β = 80.212 (5)°	Prism, colourless
γ = 78.863 (5)°	0.50 × 0.50 × 0.30 mm
*V* = 572.24 (6) Å^3^	

### Data collection

**Table:**

Oxford Diffraction Xcalibur Sapphire2 diffractometer	2017 independent reflections
Radiation source: Enhance (Mo) X-ray Source	1731 reflections with *I* > 2σ(*I*)
Graphite monochromator	*R*_int_ = 0.011
Detector resolution: 8.4353 pixels mm^-1^	θ_max_ = 25.0°, θ_min_ = 3.2°
ω scan	*h* = −6→7
Absorption correction: multi-scan (*CrysAlis RED*; Oxford Diffraction, 2009)	*k* = −10→10
*T*_min_ = 0.942, *T*_max_ = 1.000	*l* = −11→12
3413 measured reflections	

### Refinement

**Table:**

Refinement on *F*^2^	Primary atom site location: structure-invariant direct methods
Least-squares matrix: full	Secondary atom site location: difference Fourier map
*R*[*F*^2^ > 2σ(*F*^2^)] = 0.025	Hydrogen site location: inferred from neighbouring sites
*wR*(*F*^2^) = 0.069	H atoms treated by a mixture of independent and constrained refinement
*S* = 1.07	*w* = 1/[σ^2^(*F*_o_^2^) + (0.0424*P*)^2^] where *P* = (*F*_o_^2^ + 2*F*_c_^2^)/3
2017 reflections	(Δ/σ)_max_ < 0.001
170 parameters	Δρ_max_ = 0.21 e Å^−^^3^
163 restraints	Δρ_min_ = −0.33 e Å^−^^3^

### Special details

**Table:**

Experimental. IR (KBr, cm^-1^): 3298, 2924, 2852, 1441, 1296, 1237, 1189, 1096, 1043, 926, 800, 644.
Geometry. All e.s.d.'s (except the e.s.d. in the dihedral angle between two l.s. planes) are estimated using the full covariance matrix. The cell e.s.d.'s are taken into account individually in the estimation of e.s.d.'s in distances, angles and torsion angles; correlations between e.s.d.'s in cell parameters are only used when they are defined by crystal symmetry. An approximate (isotropic) treatment of cell e.s.d.'s is used for estimating e.s.d.'s involving l.s. planes.
Refinement. Refinement of *F*^2^ against ALL reflections. The weighted *R*-factor *wR* and goodness of fit *S* are based on *F*^2^, conventional *R*-factors *R* are based on *F*, with *F* set to zero for negative *F*^2^. The threshold expression of *F*^2^ > σ(*F*^2^) is used only for calculating *R*-factors(gt) *etc*. and is not relevant to the choice of reflections for refinement. *R*-factors based on *F*^2^ are statistically about twice as large as those based on *F*, and *R*- factors based on ALL data will be even larger.

### Fractional atomic coordinates and isotropic or equivalent isotropic displacement parameters (Å^2^)

**Table:**

	*x*	*y*	*z*	*U*_iso_*/*U*_eq_	Occ. (<1)
S1	0.70121 (6)	−0.24913 (4)	0.59384 (4)	0.02256 (13)	
P1	0.79402 (5)	−0.18144 (4)	0.39881 (4)	0.01557 (12)	
O1	0.71651 (15)	−0.27186 (11)	0.31916 (10)	0.0220 (3)	
O2	1.03994 (14)	−0.21705 (11)	0.36207 (10)	0.0185 (2)	
N1	0.7238 (2)	0.00511 (14)	0.31544 (13)	0.0186 (3)	
C1	0.7583 (3)	−0.44339 (18)	0.36569 (19)	0.0353 (4)	
H1B	0.6703	−0.4816	0.3219	0.053*	
H1C	0.7260	−0.4884	0.4659	0.053*	
H1D	0.9068	−0.4763	0.3407	0.053*	
C2	1.1800 (2)	−0.15705 (18)	0.41653 (17)	0.0270 (4)	
H2A	1.3260	−0.1927	0.3884	0.041*	
H2B	1.1550	−0.1971	0.5173	0.041*	
H2C	1.1541	−0.0407	0.3809	0.041*	
C3A	0.7713 (2)	0.08687 (16)	0.16597 (15)	0.0193 (3)	
H3A	0.8465	0.0061	0.1261	0.023*	0.528 (5)
C4A	0.5781 (5)	0.1703 (6)	0.0909 (3)	0.0248 (9)	0.528 (5)
H4A1	0.4971	0.2458	0.1332	0.030*	0.528 (5)
H4A2	0.4873	0.0906	0.1020	0.030*	0.528 (5)
C5A	0.6336 (6)	0.2606 (7)	−0.0631 (4)	0.0305 (12)	0.528 (5)
H5A1	0.7026	0.1841	−0.1076	0.037*	0.528 (5)
H5A2	0.5031	0.3161	−0.1064	0.037*	0.528 (5)
C6A	0.7785 (8)	0.3804 (5)	−0.0864 (4)	0.0309 (11)	0.528 (5)
H6A1	0.7043	0.4631	−0.0501	0.037*	0.528 (5)
H6A2	0.8168	0.4328	−0.1865	0.037*	0.528 (5)
C7A	0.9772 (7)	0.3018 (4)	−0.0158 (4)	0.0251 (9)	0.528 (5)
H7A1	1.0658	0.3835	−0.0290	0.030*	0.528 (5)
H7A2	1.0585	0.2260	−0.0578	0.030*	0.528 (5)
C8A	0.9216 (7)	0.2137 (5)	0.1383 (4)	0.0235 (9)	0.528 (5)
H8A1	0.8522	0.2914	0.1814	0.028*	0.528 (5)
H8A2	1.0524	0.1596	0.1818	0.028*	0.528 (5)
H3B	0.8963	0.0240	0.1326	0.023*	0.472 (5)
C4B	0.5791 (6)	0.0888 (6)	0.0963 (4)	0.0202 (9)	0.472 (5)
H4B1	0.5555	−0.0214	0.1169	0.024*	0.472 (5)
H4B2	0.4515	0.1442	0.1329	0.024*	0.472 (5)
C5B	0.6199 (7)	0.1738 (6)	−0.0588 (4)	0.0237 (10)	0.472 (5)
H5B1	0.7365	0.1106	−0.0964	0.028*	0.472 (5)
H5B2	0.4929	0.1831	−0.1025	0.028*	0.472 (5)
C6B	0.6762 (10)	0.3388 (6)	−0.0932 (5)	0.0257 (12)	0.472 (5)
H6B1	0.7101	0.3888	−0.1940	0.031*	0.472 (5)
H6B2	0.5536	0.4057	−0.0646	0.031*	0.472 (5)
C7B	0.8621 (10)	0.3311 (6)	−0.0215 (5)	0.0299 (11)	0.472 (5)
H7B1	0.8908	0.4402	−0.0426	0.036*	0.472 (5)
H7B2	0.9880	0.2730	−0.0569	0.036*	0.472 (5)
C8B	0.8214 (9)	0.2479 (5)	0.1350 (4)	0.0250 (10)	0.472 (5)
H8B1	0.9475	0.2403	0.1789	0.030*	0.472 (5)
H8B2	0.7026	0.3100	0.1722	0.030*	0.472 (5)
H1N	0.634 (2)	0.0509 (18)	0.3521 (16)	0.021 (4)*	

### Atomic displacement parameters (Å^2^)

**Table:**

	*U*^11^	*U*^22^	*U*^33^	*U*^12^	*U*^13^	*U*^23^
S1	0.0223 (2)	0.0192 (2)	0.0187 (2)	0.00165 (15)	0.00233 (15)	−0.00272 (16)
P1	0.0129 (2)	0.0147 (2)	0.0178 (2)	0.00016 (14)	−0.00189 (15)	−0.00541 (16)
O1	0.0211 (5)	0.0169 (5)	0.0302 (6)	0.0007 (4)	−0.0100 (5)	−0.0095 (5)
O2	0.0130 (5)	0.0207 (5)	0.0226 (6)	−0.0006 (4)	−0.0017 (4)	−0.0097 (4)
N1	0.0186 (6)	0.0164 (7)	0.0174 (7)	0.0025 (5)	0.0009 (5)	−0.0060 (5)
C1	0.0376 (10)	0.0200 (9)	0.0541 (12)	0.0017 (7)	−0.0183 (9)	−0.0170 (8)
C2	0.0171 (8)	0.0286 (9)	0.0380 (10)	−0.0048 (6)	−0.0065 (7)	−0.0126 (8)
C3A	0.0214 (8)	0.0181 (8)	0.0163 (8)	0.0006 (6)	−0.0008 (6)	−0.0059 (6)
C4A	0.0235 (16)	0.030 (2)	0.0192 (16)	−0.0038 (17)	−0.0037 (12)	−0.0069 (17)
C5A	0.026 (2)	0.044 (4)	0.0176 (19)	−0.005 (3)	−0.0063 (15)	−0.005 (2)
C6A	0.031 (3)	0.026 (2)	0.023 (2)	−0.0014 (19)	0.0021 (18)	0.0004 (16)
C7A	0.027 (2)	0.0221 (18)	0.0242 (18)	−0.0088 (17)	0.0028 (18)	−0.0068 (14)
C8A	0.026 (2)	0.021 (2)	0.0226 (17)	−0.0055 (17)	−0.0026 (18)	−0.0059 (14)
C3B	0.0214 (8)	0.0181 (8)	0.0163 (8)	0.0006 (6)	−0.0008 (6)	−0.0059 (6)
C4B	0.0229 (17)	0.017 (2)	0.0189 (18)	−0.0018 (18)	−0.0045 (13)	−0.0045 (17)
C5B	0.031 (2)	0.021 (2)	0.0209 (18)	−0.0037 (18)	−0.0053 (14)	−0.0081 (18)
C6B	0.038 (3)	0.017 (3)	0.018 (2)	−0.005 (2)	−0.003 (2)	−0.0017 (19)
C7B	0.037 (3)	0.024 (2)	0.028 (2)	−0.015 (2)	−0.004 (2)	−0.0028 (18)
C8B	0.031 (3)	0.021 (2)	0.0230 (19)	−0.007 (2)	−0.006 (2)	−0.0051 (16)

### Geometric parameters (Å, º)

**Table:**

S1—P1	1.9351 (5)	C6A—C7A	1.521 (5)
P1—O1	1.5823 (10)	C6A—H6A1	0.9900
P1—O2	1.5853 (9)	C6A—H6A2	0.9900
P1—N1	1.6153 (12)	C7A—C8A	1.531 (5)
O1—C1	1.4471 (18)	C7A—H7A1	0.9900
O2—C2	1.4456 (17)	C7A—H7A2	0.9900
N1—C3A	1.4739 (18)	C8A—H8A1	0.9900
N1—H1N	0.791 (16)	C8A—H8A2	0.9900
C1—H1B	0.9800	C4B—C5B	1.523 (5)
C1—H1C	0.9800	C4B—H4B1	0.9900
C1—H1D	0.9800	C4B—H4B2	0.9900
C2—H2A	0.9800	C5B—C6B	1.523 (5)
C2—H2B	0.9800	C5B—H5B1	0.9900
C2—H2C	0.9800	C5B—H5B2	0.9900
C3A—C4A	1.519 (4)	C6B—C7B	1.513 (6)
C3A—C8A	1.578 (4)	C6B—H6B1	0.9900
C3A—H3A	1.0000	C6B—H6B2	0.9900
C4A—C5A	1.533 (5)	C7B—C8B	1.534 (6)
C4A—H4A1	0.9900	C7B—H7B1	0.9900
C4A—H4A2	0.9900	C7B—H7B2	0.9900
C5A—C6A	1.512 (6)	C8B—H8B1	0.9900
C5A—H5A1	0.9900	C8B—H8B2	0.9900
C5A—H5A2	0.9900		
			
O1—P1—O2	99.23 (5)	C5A—C6A—H6A1	109.4
O1—P1—N1	105.48 (6)	C7A—C6A—H6A1	109.4
O2—P1—N1	107.01 (6)	C5A—C6A—H6A2	109.4
O1—P1—S1	115.17 (4)	C7A—C6A—H6A2	109.4
O2—P1—S1	114.80 (4)	H6A1—C6A—H6A2	108.0
N1—P1—S1	113.73 (5)	C6A—C7A—C8A	110.4 (3)
C1—O1—P1	120.01 (9)	C6A—C7A—H7A1	109.6
C2—O2—P1	119.12 (9)	C8A—C7A—H7A1	109.6
C3A—N1—P1	124.89 (10)	C6A—C7A—H7A2	109.6
C3A—N1—H1N	115.6 (11)	C8A—C7A—H7A2	109.6
P1—N1—H1N	117.5 (11)	H7A1—C7A—H7A2	108.1
O1—C1—H1B	109.5	C7A—C8A—C3A	112.0 (3)
O1—C1—H1C	109.5	C7A—C8A—H8A1	109.2
H1B—C1—H1C	109.5	C3A—C8A—H8A1	109.2
O1—C1—H1D	109.5	C7A—C8A—H8A2	109.2
H1B—C1—H1D	109.5	C3A—C8A—H8A2	109.2
H1C—C1—H1D	109.5	H8A1—C8A—H8A2	107.9
O2—C2—H2A	109.5	C5B—C4B—H4B1	109.8
O2—C2—H2B	109.5	C5B—C4B—H4B2	109.8
H2A—C2—H2B	109.5	H4B1—C4B—H4B2	108.3
O2—C2—H2C	109.5	C6B—C5B—C4B	111.1 (3)
H2A—C2—H2C	109.5	C6B—C5B—H5B1	109.4
H2B—C2—H2C	109.5	C4B—C5B—H5B1	109.4
N1—C3A—C4A	113.92 (16)	C6B—C5B—H5B2	109.4
N1—C3A—C8A	109.76 (17)	C4B—C5B—H5B2	109.4
C4A—C3A—C8A	108.4 (2)	H5B1—C5B—H5B2	108.0
N1—C3A—H3A	108.2	C7B—C6B—C5B	111.3 (4)
C4A—C3A—H3A	108.2	C7B—C6B—H6B1	109.4
C8A—C3A—H3A	108.2	C5B—C6B—H6B1	109.4
C3A—C4A—C5A	112.6 (3)	C7B—C6B—H6B2	109.4
C3A—C4A—H4A1	109.1	C5B—C6B—H6B2	109.4
C5A—C4A—H4A1	109.1	H6B1—C6B—H6B2	108.0
C3A—C4A—H4A2	109.1	C6B—C7B—C8B	111.5 (4)
C5A—C4A—H4A2	109.1	C6B—C7B—H7B1	109.3
H4A1—C4A—H4A2	107.8	C8B—C7B—H7B1	109.3
C6A—C5A—C4A	111.4 (3)	C6B—C7B—H7B2	109.3
C6A—C5A—H5A1	109.3	C8B—C7B—H7B2	109.3
C4A—C5A—H5A1	109.3	H7B1—C7B—H7B2	108.0
C6A—C5A—H5A2	109.3	C7B—C8B—H8B1	109.8
C4A—C5A—H5A2	109.3	C7B—C8B—H8B2	109.8
H5A1—C5A—H5A2	108.0	H8B1—C8B—H8B2	108.3
C5A—C6A—C7A	111.3 (3)		

### Hydrogen-bond geometry (Å, º)

**Table:**

*D*—H···*A*	*D*—H	H···*A*	*D*···*A*	*D*—H···*A*
N1—H1*N*···S1^i^	0.791 (16)	2.695 (17)	3.4633 (13)	164.3 (14)

Symmetry code: (i) −*x*+1, −*y*, −*z*+1.
